# Institutional enrollment volume and clinical outcomes after surgery or chemoradiotherapy for clinical stage I esophageal squamous cell carcinoma: a supplementary analysis of JCOG0502

**DOI:** 10.1007/s10388-026-01244-w

**Published:** 2026-08-27

**Authors:** Kazuhiro Shiraishi, Ken Kato, Yuya Ishikawa, Keita Sasaki, Hiroyuki Daiko, Yoshinori Ito, Takeshi Kajiwara, Takashi Kojima, Hiroshi Miyata, Masaki Ueno, Satoru Nakagawa, Shinsuke Sato, Shigeru Tsunoda, Tetsuya Abe, Yoshiaki Nagatani, Yoichi Hamai, Yasue Kimura, Kenro Hirata, Takahiro Tsushima, Hiroya Takeuchi

**Affiliations:** 1 Department of Head and Neck, Esophageal Medical Oncology, National Cancer Center Hospital, 5-1-1 Tsukiji, Chuo-ku, Tokyo, 104-0045 Japan; 2 JCOG Data Center/Operations Office, National Cancer Center Hospital, Chuo City, Japan; 3 Department of Esophageal Surgery, National Cancer Center Hospital, Chuo City, Japan; 4 Department of Radiation Oncology, Showa Medical University School of Medicine, Tokyo, Japan; 5 Department of Gastrointestinal Medical Oncology, National Hospital Organization Shikoku Cancer Center, Matsuyama, Japan; 6 Division of Gastrointestinal Oncology, National Cancer Center Hospital East, Kashiwa, Japan; 7 Department of Gastroenterological Surgery, Osaka International Cancer Institute, Osaka, Japan; 8 Department of Gastroenterological Surgery, Toranomon Hospital, Minato, Japan; 9 Department of Gastroenterological Surgery, Niigata Cancer Center Hospital, Niigata, Japan; 10 Department of Gastroenterological Surgery, Shizuoka General Hospital, Shizuoka, Japan; 11 Department of Surgery, Kyoto University, Kyoto, Japan; 12 Department of Gastroenterological Surgery, Aichi Cancer Center Hospital, Nagoya, Japan; 13 Department of Medical Oncology and Hematology, Kobe University Hospital, Kobe, Japan; 14 Department of Surgical Oncology, Hiroshima University, Higashihiroshima, Japan; 15 Department of Gastrointestinal Surgery, NHO Kyushu Cancer Center, Fukuoka, Japan; 16 Division of Gastroenterology and Hepatology, Department of Internal Medicine, Keio University School of Medicine, Tokyo, Japan; 17 Division of Gastrointestinal Oncology, Shizuoka Cancer Center, Shizuoka, Japan; 18 Department of Surgery, Hamamatsu University School of Medicine, Hamamatsu, Japan

**Keywords:** Esophageal squamous cell carcinoma, Chemoradiotherapy, Surgery, JCOG0502, Institutional heterogeneity

## Abstract

**Background:**

Surgery and chemoradiotherapy (CRT) are standard options for clinical stage I esophageal squamous cell carcinoma (ESCC). JCOG0502, a multicenter, non-randomized, parallel-group comparison, demonstrated the non-inferiority of definitive CRT compared with surgery for overall survival (OS) in patients with cT1bN0M0 ESCC; however, institutional differences may influence outcomes. This study explored inter-institutional heterogeneity in survival and adverse events (AEs) among patients in JCOG0502.

**Methods:**

Among 379 enrolled patients, 364 were analyzed. Institutions enrolling ≥ 13 patients, corresponding to the median institutional trial enrollment, were classified as high-enrollment institutions (High), whereas those enrolling < 13 patients were classified as low-enrollment institutions (Low). Institutional trial enrollment was evaluated as an exploratory indicator, rather than as a direct measure of institutional case or procedural volume. The primary endpoint was OS. Secondary endpoints were progression-free survival (PFS) and AEs.

**Results:**

In the surgery group, institutional trial enrollment volume was not significantly associated with OS (HR for High vs. Low, 0.73; 95% CI, 0.40–1.35). Similarly, no significant association was observed in the CRT group (HR, 1.12; 95% CI, 0.59–2.13). Findings for PFS were consistent with these OS results in both groups. Myocardial ischemia or infarction occurred in 5 patients in Low and none in High in the CRT group.

**Conclusions:**

No clear association was identified between institutional trial enrollment volume and survival outcomes in either the surgery or CRT group. However, clinically meaningful differences could not be excluded. The observed differences in selected toxicities, including myocardial ischemia, should be considered hypothesis-generating.

**Supplementary Information:**

The online version contains supplementary material available at https://doi.org/10.1007/s10388-026-01244-w.

## Introduction

Esophageal squamous cell carcinoma (ESCC) remains a major global health burden, particularly in East Asia, where squamous histology predominates. Japan has one of the highest incidences of ESCC worldwide, and early-stage disease is increasingly detected through surveillance and screening programs [1, 2].

For patients with clinical stage I ESCC (cT1bN0M0), both esophagectomy with lymphadenectomy and definitive chemoradiotherapy (CRT) are established curative treatment options according to Japanese and international guidelines [3, 4]. The multicenter non-randomized parallel group comparison, JCOG0502, demonstrated the non-inferiority of definitive CRT compared with surgery in terms of overall survival [OS] among patients with cT1bN0M0 ESCC [5]. This result established definitive CRT as a standard alternative to surgery in appropriately selected patients in Japan. Notably, salvage treatment, including surgery, chemotherapy, or endoscopic resection, was allowed after recurrence, reflecting a multimodal treatment strategy.

Although JCOG trials incorporate strict protocol standardization, credentialing, and central monitoring, inter-institutional differences in treatment selection and delivery may still exist: treatment decision-making in esophageal cancer is influenced by institutional expertise and physician recommendations, which in turn shape patient preferences [6, 7]. However, the extent to which such institutional tendencies translate into differences in survival outcomes or adverse events [AEs] has not been adequately investigated.

In surgical management, a consistent volume–outcome relationship has been reported, with high-volume centers demonstrating lower postoperative mortality and improved long-term survival [8, 9]. Similar concerns have been raised about the quality of radiotherapy: protocol deviations such as target volume delineation errors and dose deviations have been associated with inferior survival outcomes in cooperative group trials [10, 11], and cardiac radiation dose has been linked to both cardiac morbidity and overall survival after CRT [12]. Institutional trial accrual volume, distinct from institutional case volume, has previously been used as an indicator of institutional experience with cooperative group research: higher institutional accrual to prior Radiation Therapy Oncology Group trials has been associated with improved survival among patients with head and neck cancer and non-small-cell lung cancer treated with chemoradiotherapy, an association partly attributed to differences in radiotherapy protocol compliance [13, 14]. In contrast, no association between institutional accrual volume and survival was observed among patients with non-small-cell lung cancer treated with chemoradiotherapy in a trial with rigorous radiotherapy quality assurance [15], suggesting that the relationship between trial accrual volume and outcomes may depend on the intensity of protocol-driven quality control. Given the increasing adoption of definitive CRT for stage I ESCC, understanding potential institutional variability is clinically relevant.

Therefore, this exploratory supplementary analysis of JCOG0502 aimed to examine whether institutional trial enrollment volume was associated with survival and safety outcomes. Institutional trial enrollment was evaluated as an indicator of institutional participation in and experience with protocol-driven cooperative group research, rather than as a direct measure of institutional esophageal cancer case volume, surgical volume, radiotherapy volume, or treatment quality.

## Materials and methods

### Study design and patients

JCOG0502 was a parallel-group controlled trial conducted by the Japan Esophageal Oncology Group [5]. The trial enrolled patients with clinical stage I ESCC (cT1bN0M0, UICC 6th edition) between 2006 and 2013. Eligible patients were either randomized or self-selected to undergo surgery or definitive CRT, based on the institution’s enrollment strategy (UMIN000000551).

A total of 364 patients were analyzed after excluding 15 patients from the 379 enrolled patients: 2 who received non-protocol treatment and 13 who were ineligible. A total of 364 patients (202 in the surgery group, 162 in the CRT group) from 37 participating institutions were eligible. Institutional trial enrollment varied substantially across these institutions. The median institutional enrollment was 13 patients, with a range of 1–71 and an interquartile range of 7–26. The distribution of enrollment across institutions is shown in Supplementary Figure S1. Institutional identifiers were anonymized. Baseline characteristics and surgical findings were analyzed in all 202 patients. However, efficacy and safety analyses were conducted in 201 patients, excluding one patient who withdrew consent after enrollment.

### Treatment

In the surgery group, right transthoracic esophagectomy with D2 or more extensive lymphadenectomy was required. Either thoracotomy or thoracoscopic surgery and either two-field or three-field lymphadenectomy were permitted according to the protocol.

The CRT group received 60 Gy in 30 fractions (2 Gy per fraction, 5 fractions per week; planned treatment time, 42 days), concurrent with 5-fluorouracil (700 mg/m² on days 1–4 and 29–32) and cisplatin (70 mg/m² on days 1 and 29). Radiotherapy target volumes were defined according to the study protocol, and prophylactic (elective) nodal irradiation was not permitted. Protocol-specified dose constraints for organs at risk included a maximum spinal cord dose of 44 Gy and a maximum whole-heart dose of 40 Gy, with the irradiated cardiac volume minimized as much as possible; no formal dose limit was specified for the lung, but the volume receiving ≥ 20 Gy (V20) was to be minimized. Further details of the radiotherapy equipment, target volume definitions, dose calculation, and treatment verification procedures are provided in Supplementary Methods. Salvage surgery, chemotherapy, and endoscopic mucosal resection/endoscopic submucosal dissection were permitted for local recurrence or persistent disease.

### Statistical analysis

The primary endpoint was OS, defined as the time from enrollment to death from any cause, and was censored at the last contact with a surviving patient. Secondary endpoints included progression-free survival (PFS) and AEs. Progression was defined by imaging or clinical deterioration. PFS was defined as the time from enrollment to disease progression or death by any cause, or was censored at the last date of confirmed survival without disease progression. The AEs were evaluated according to the Common Terminology Criteria for Adverse Events version 3.0, a Japanese translation of the JCOG version. Acute CRT-related AEs were defined as those occurring from the start of protocol treatment to 30 days after its completion or termination, while late CRT-related AEs were defined as those occurring from 31 days after completion or termination.

Institutional trial enrollment was defined as the number of eligible patients included in the present analysis from each participating institution. Because enrollment varied substantially across institutions, several exploratory thresholds were examined during the planning of this supplementary analysis, including 5 patients, 10 patients, and the median institutional enrollment of 13 patients. The median cut-off was selected for the main analysis because it provided a more balanced distribution of patients between the enrollment groups and avoided the very small low-enrollment group generated by lower thresholds. Institutions enrolling ≥ 13 patients were classified as high-enrollment institutions (High), whereas those enrolling < 13 patients were classified as low-enrollment institutions (Low). This classification included 9 high-enrollment institutions and 28 low-enrollment institutions. As a sensitivity analysis, institutional trial enrollment was also evaluated as a continuous variable in Cox proportional hazards models. Hazard ratios were estimated per five-patient increase in institutional enrollment. The OS and PFS were estimated using the Kaplan-Meier method. The hazard ratio [HR] for High vs. Low, along with its 95% confidence interval [CI] and p-value, was estimated using the Cox proportional hazards model. Differences between the Low and High groups were compared using the Wilcoxon rank-sum test for continuous variables and Fisher’s exact test for categorical variables.

All p-values were two-sided, and a p-value of < 0.05 was considered statistically significant; however, no adjustment for multiplicity was performed, and the results should be interpreted as exploratory. SAS version 9.4 statistical software was used.

## Results

### Patient characteristics

Among the 37 institutions included in the analysis, 28 were classified as Low and 9 as High. The median institutional trial enrollment was 13 patients (IQR, 7–26; range, 1–71). The distribution of institutional enrollment is shown in Supplementary Figure S1. A total of 202 patients (*n* = 98: Low, *n* = 104: High) in the surgery group and 162 patients (*n* = 59: Low, *n* = 103: High) in the CRT group were analyzed. Baseline patient characteristics were generally comparable between Low and High institutions in both the surgery and CRT groups, although several institution-dependent surgical practice variables differed between them. (Table 1).

**Table 1:** Baseline characteristics according to institutional trial enrollment volume

Characteristics	Surgery (*n* = 202)			Chemoradiotherapy (*n* = 162)		
	< 13 patients (Low) (*n* = 98)	≥ 13 patients (High) (*n* = 104)	*P* value*	< 13 patients (Low) (*n* = 59)	≥ 13 patients (High) (*n* = 103)	*P* value*
Age (years)	61.5	62	0.85	65	65	0.63
Median, (range)	(46–75)	(41–75)		(49–75)	(42–75)	
Sex						
Male	84	84	0.45	50	92	0.46
Female	14	20		9	11	
Performance status						
0	97	104	0.49	57	102	0.30
1	1	0		2	1	
Primary tumor location						
Upper thoracic (Ut)	13	12	0.96	4	10	0.82
Middle thoracic (Mt)	61	67		36	63	
Lower thoracic (Lt)	24	25		19	30	
Histological diagnosis						
Squamous cell carcinoma	97	104	0.49	59	103	-
Well differentiated	8	5		9	1	
Moderately differentiated	37	26		25	34	
Poorly differentiated	10	10		6	4	
Differentiation unknown	42	63		19	64	
Basaloid carcinoma	1	0		0	0	
Multiple lesions (satellite lesions)						
Absent	85	98	0.09	50	88	1.00
Present	13	6		9	15	
Tumor length						
≤ 4 cm	70	71	0.65	37	64	1.00
> 4 cm	28	33		22	39	
Body mass index						
Median, (Range)	22.3 (16.4–28.9)	22.6 (17.1–28.4)	0.85	23 (17.8–27.7)	22.5 (14.7–27.7)	0.43

Two-sided p-values were calculated using the Wilcoxon rank-sum test for continuous variables and Fisher’s exact test for categorical variables

### Efficacy

**Fig. 1:**
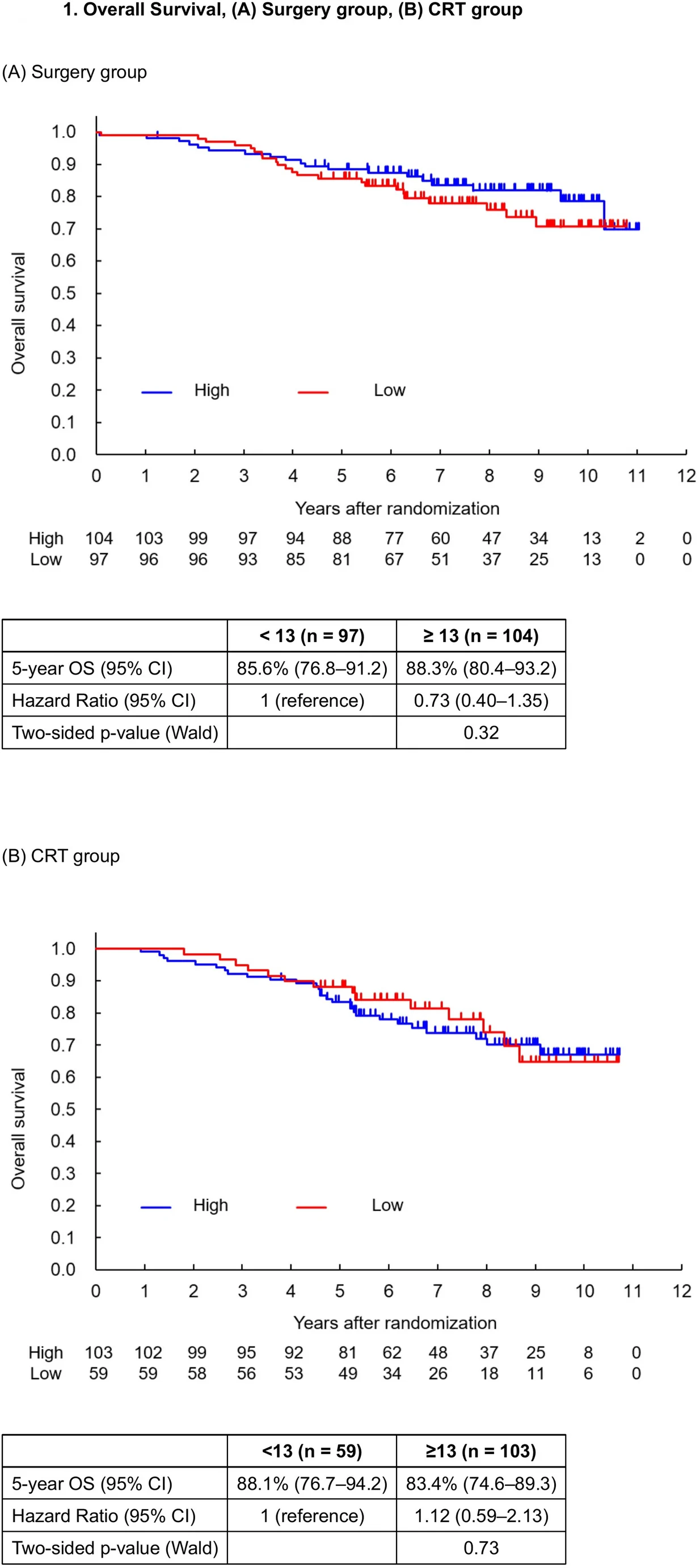
Overall survival, **A** Surgery group, **B** CRT group. *OS* overall survival, *CI* confidence interval, *CRT* chemoradiotherapy

**Fig. 2:**
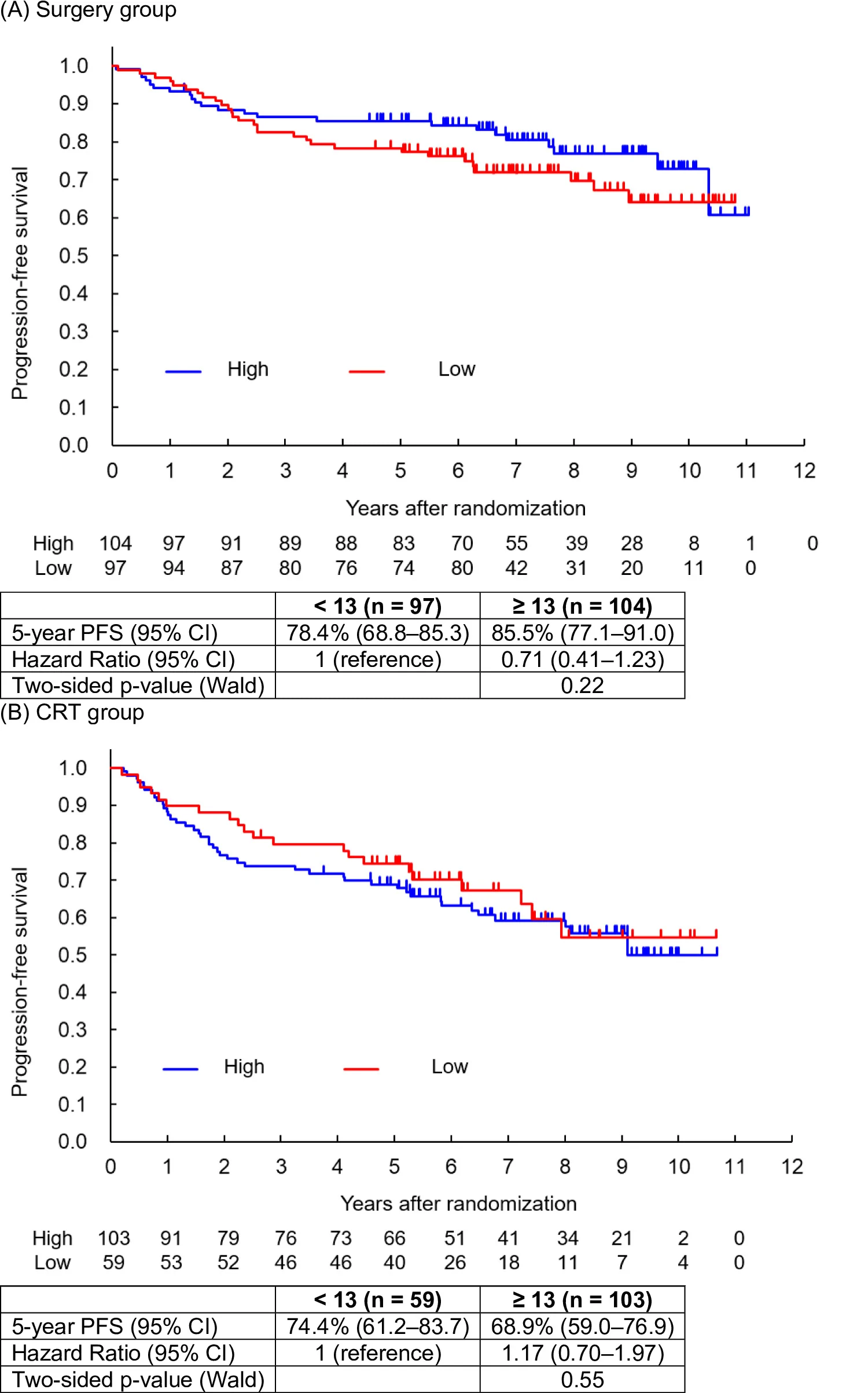
Progression-free survival **A** Surgery group, **B** CRT group. *PFS* progression-free survival, *CI* confidence interval, *CRT* chemoradiotherapy

Median follow-up was 7.0 years. The 5-year OS (Low vs. High) was 85.6% (95% CI: 76.8–91.2) vs. 88.3% (95% CI: 80.4–93.2) in the surgery group (HR, 0.73; 95% CI, 0.40–1.35; *p* = 0.32) and 88.1% (95% CI: 76.7–94.2) vs. 83.4% (95% CI: 74.6–89.3) in the CRT group (HR, 1.12; 95% CI, 0.59–2.13; *p* = 0.73) (Fig. 1A, B). The 5-year PFS (Low vs. High) was 78.4% (95% CI: 68.8–85.3) vs. 85.5% (95% CI: 77.1–91.0) in the surgery group (HR, 0.71; 95% CI, 0.41–1.23; *p* = 0.22) and 74.4% (95% CI: 61.2–83.7) vs. 68.9% (95% CI: 59.0–76.9) in the CRT group (HR, 1.17; 95% CI, 0.70–1.97; *p* = 0.55) (Fig. 2A, B). No statistically significant association was identified between the median-based institutional trial enrollment category and OS or PFS in either treatment group. However, the wide confidence intervals indicate that clinically meaningful differences could not be excluded. When institutional trial enrollment was analyzed as a continuous variable, no significant association was observed with OS or PFS in either the surgery or CRT group (Supplementary Table S2).

### Surgical procedures and pathological findings

Details of the surgical findings are presented in Table 2. The abdominal approach differed between enrollment groups. Open laparotomy was performed more frequently in High than in Low (75.0% vs. 60.2%, *p* = 0.03). A marked difference was observed in the reconstruction route (Antethoracic/Retrosternal/Posterior mediastinal/Intrathoracic (right): 5/17/68/8 vs. 0/51/49/4, *p* < 0.01). The posterior mediastinal route was more commonly selected in Low, whereas the retrosternal route was more frequently used in High. The extent of lymphadenectomy also differed significantly between groups (D1/D2/D3/Missing: 0/60/37/1 vs. 2/47/55/0, *p* = 0.02). D3 dissection was performed more often in High, whereas D2 dissection was more common in Low. No significant differences were observed in thoracic approach, pathological stage, or the total number of retrieved lymph nodes (all *p* > 0.05). These differences likely reflected institution-specific surgical practice patterns and should be considered when interpreting the unadjusted survival comparisons.

**Table 2:** Surgical procedures and pathological findings according to institutional trial enrollment volume

	Total (*n* = 202)	< 13 patients (Low) (*n* = 98)	≥ 13 patients (High) (*n* = 104)	*P* value*
Abdominal approach				
Open laparotomy	137	59	78	0.03
Laparoscopy-assisted	64	38	26	
Missing	1	1	0	
Thoracic approach				
Open thoracotomy	103	52	51	0.58
Thoracoscopic	99	46	53	
Reconstruction route				
Antethoracic	5	5	0	**< 0.01**
Retrosternal	68	17	51	
Posterior mediastinal	117	68	49	
Intrathoracic (right)	12	8	4	
Intrathoracic (left)	0	0	0	
Other	0	0	0	
Pathological stage (UICC)				
p-0	4	1	3	0.74
p-I	141	70	71	
p-IIA	3	1	2	
p-IIB	43	22	21	
p-III	1	0	1	
p-IV	0	0	0	
p-IVA	0	0	0	
p-IVB	9	3	6	
Missing	1	1	0	
Total number of retrieved lymph nodes				
Median	52	50	52.5	0.20
Interquartile range (P25–P75)	39–70	37–66	39.5–74	
Range (min–max)	18–120	19–104	18–120	
Extent of lymphadenectomy				
D0	0	0	0	**0.02**
D1	2	0	2	
D2	107	60	47	
D3	92	37	55	
Missing	1	1	0	

Bold values indicate statistical significance (p < 0.05)

### Safety

#### Postoperative complications

Postoperative complications were analyzed in 201 eligible patients who underwent protocol surgery after excluding one patient who withdrew consent. No significant inter-institutional differences in major postoperative complications were observed between Low (*n* = 97) and High (*n* = 104). The incidence of gastrointestinal fistula was comparable between Low and High (Low vs. High) for both all-grade events and grade ≥ 3 events (all-grade: 1.0% vs. 2.9%, *p* = 0.62; grade ≥ 3: 1.0% vs. 2.9%, *p* = 0.62). Similarly, no significant differences were observed in the proportions of anastomotic leak (all-grade: 15.5% vs. 10.6%, *p* = 0.40; grade ≥ 3: 9.3% vs. 3.9%, *p* = 0.15), atelectasis (all-grade: 23.7% vs. 22.1%, *p* = 0.87; grade ≥ 3: 3.1% vs. 1.0%, *p* = 0.35), or recurrent laryngeal nerve palsy (all-grade: 20.6% vs. 19.2%, *p* = 0.86; grade ≥ 3: 2.1% vs. 3.9%, *p* = 0.68). Overall, postoperative morbidity did not differ significantly between Low and High with different enrollment volumes. (Table 3).

**Table 3:** Early postoperative complications according to institutional trial enrollment volume

Complications	All patients *n* = 201 (%)		< 13 patients (Low) *n* = 97 (%)		≥ 13 patients (High) *n* = 104 (%)		*P* value*
	All Grade	Grade ≥ 3	All Grade	Grade ≥ 3	All Grade	Grade ≥ 3	
Gastrointestinal fistula	4 (2.0)	4 (2.0)	1 (1.0)	1 (1.0)	3 (2.9)	3 (2.9)	0.62 0.62
Gastrointestinal leak	26 (12.9)	13 (6.5)	15 (15.5)	9 (9.3)	11 (10.6)	4 (3.9)	0.40 0.15
Atelectasis	46 (22.9)	4 (2.0)	23 (23.7)	3 (3.1)	23 (22.1)	1 (1.0)	0.86 0.35
Recurrent laryngeal nerve palsy	40 (19.9)	1 (3.0)	20 (20.6)	2 (2.1)	20 (19.2)	4 (3.9)	0.86 0.68

Two-sided p-values were calculated using Fisher’s exact test

*The upper P value corresponds to all-grade events, and the lower P value corresponds to grade ≥ 3 events

#### Adverse Events in the Chemoradiotherapy Cohort

A total of 162 eligible patients who received definitive CRT were analyzed (Low: *n* = 59; High: *n* = 103). Among acute AEs, significant inter-institutional differences were observed in the incidence of esophagitis and radiation dermatitis. All-grade esophagitis occurred more frequently in High than in Low (91.3% vs. 76.3%, *p* = 0.01), although the incidence of grade ≥ 3 events did not differ significantly (7.8% vs. 13.6%, *p* = 0.28). Radiation dermatitis was more common in Low (22.0% vs. 9.7%, *p* = 0.04), with no grade ≥ 3 events reported in either group. No significant differences were observed in hematologic toxicities, including leukopenia, neutropenia, thrombocytopenia, or anemia (all *p* > 0.05). (Table 4).

**Table 4:** Acute adverse events in the chemoradiotherapy group according to institutional trial enrollment volume

Adverse Events	All patients *n* = 162 (%)		< 13 patients (Low) *n* = 59 (%)		≥ 13 patients (High) *n* = 103 (%)		*P* value*
	All Grade	Grade ≥ 3	All Grade	Grade ≥ 3	All Grade	Grade ≥ 3	
Leukopenia	154 (95.1)	19 (11.7)	58 (98.3)	6 (10.2)	96 (93.2)	13 (12.6)	0.26 0.80
Neutropenia	37 (22.8)	18 (11.1)	48 (81.4)	8 (13.6)	84 (81.6)	10 (9.7)	1.00 0.45
Thrombocytopenia	37 (22.8)	2 (1.2)	14 (23.7)	0	23 (22.3)	2 (1.9)	0.85 0.53
Esophagitis	139 (85.8)	16 (9.9)	45 (76.3)	8 (13.6)	94 (91.3)	8 (7.8)	**0.01** 0.28
Nausea	99 (61.1)	7 (4.3)	31 (52.5)	4 (6.8)	68 (66.0)	3 (2.9)	0.10 0.26
Vomiting	27 (16.7)	0	12 (20.3)	0	15 (14.6)	0	0.38 –
Oral mucositis	48 (29.6)	0	23 (39.0)	0	25 (24.3)	0	0.05 –
Infection with neutropenia (any grade)	5 (3.1)	2 (1.2)	4 (6.8)	2 (3.4)	1 (1.0)	0	0.06 0.13
Radiation dermatitis	23 (14.2)	0	13 (22.0)	0	10 (9.7)	0	**0.04** –
AST elevation	38 (23.5%)	0	18 (30.5%)	0	20 (19.4)	0	0.13 –
ALT elevation	67 (41.4%)	0	27 (45.8%)	0	40 (38.8)	1 (1.0)	0.41 1.00
Anemia	60 (37.0%)	2 (1.2%)	25 (42.4%)	1 (1.7)	35 (34.0)	1 (1.0)	0.31 1.00
Creatinine increase	68 (42.0%)	0	27 (45.8%)	0	41 (39.8)	0	0.51 –

*CTCAE* common terminology criteria for adverse events, *AST* aspartate aminotransferase, *ALT* alanine aminotransferase

Two-sided p-values were calculated using Fisher’s exact test

*The upper P value corresponds to all-grade events, and the lower P value corresponds to grade ≥ 3 events

Bold values indicate statistical significance (p < 0.05)

Among late toxicities, a statistically significant difference was observed in cardiac ischemic events. Myocardial ischemia or infarction occurred in 5 patients in Low and no patients in High (8.5% vs. 0%; *p* < 0.01 for both all-grade and grade ≥ 3 events). No significant inter-institutional differences were detected in late esophagitis, dyspnea, pneumonitis, pleural effusion, or pericardial effusion (all *p* > 0.05). (Table 5).

**Table 5:** Late toxicities in the chemoradiotherapy group according to institutional trial enrollment volume

Adverse Event (CTCAE)	All patients *n* = 162 (%)		< 13 patients (Low) *n* = 59 (%)		≥ 13 patients (High) *n* = 103 (%)		*P* value*
	All Grade	Grade ≥ 3	All Grade	Grade ≥ 3	All Grade	Grade ≥ 3	
Esophagitis	36 (22.2)	1 (0.6)	16 (27.1)	1 (1.7)	20 (19.4)	0	0.33 0.36
Dyspnea	9 (5.6)	4 (2.5)	4 (6.8)	3 (5.1)	5 (4.9)	1 (1.0)	0.72 0.14
Pneumonitis	53 (32.7)	3 (1.9)	14 (23.7)	2 (3.4)	39 (37.9)	1 (1.0)	0.08 0.30
Pleural effusion	21 (13.0)	4 (2.5)	7 (11.9)	2 (3.4)	14 (13.6)	2 (1.9)	0.81 0.62
Myocardial ischemia / infarction	5 (3.1)	5 (3.1)	5 (8.5)	5 (8.5)	0	0	**< 0.01** **< 0.01**
Pericardial effusion	27 (16.7)	0	13 (22.0)	0	14 (13.6)	0	0.19 –

*CTCAE* common terminology criteria for adverse events

Two-sided p-values were calculated using Fisher’s exact test

*The upper P value corresponds to all-grade events, and the lower P value corresponds to grade ≥ 3 events

Bold values indicate statistical significance (p < 0.05)

## Discussion

In this exploratory supplementary analysis of JCOG0502, no statistically significant association was identified between institutional trial enrollment volume and OS or PFS in either the surgery or CRT group. However, this analysis was not designed or powered to establish equivalence between institutions, and the wide confidence intervals do not exclude clinically meaningful differences. Several surgical practice variables and selected treatment-related toxicities differed between enrollment groups, indicating that some institutional heterogeneity remained within the standardized cooperative group trial setting.

Institutional trial enrollment volume should not be interpreted as equivalent to overall institutional esophageal cancer case volume, surgical volume, radiotherapy volume, or treatment quality. Enrollment may be influenced by the duration of trial participation, institutional treatment preferences, referral patterns, patient choice, and research infrastructure. In the present study, trial enrollment was used only as an exploratory indicator that may reflect institutional engagement in cooperative group research, familiarity with protocol-driven multidisciplinary management, and the capacity to enroll and manage patients within a structured trial framework. Because this relationship is indirect, the present results should be interpreted as hypothesis-generating. The absence of an association in the present analysis contrasts with prior reports of a positive association between institutional trial accrual volume and survival among patients treated with chemoradiotherapy for head and neck cancer and non-small-cell lung cancer, in which the association was attributed in part to differences in radiotherapy protocol compliance across institutions [13, 14]. It is more consistent with a report in which no such association was observed within a trial incorporating rigorous radiotherapy quality assurance [15]. This contrast suggests that the extent to which institutional trial accrual volume reflects meaningful differences in outcomes may depend on the degree of protocol standardization and quality control within a given cooperative group trial, rather than on accrual volume itself.

The median institutional enrollment of 13 patients was used for the main analysis because it yielded a more balanced comparison than lower thresholds. Analyses using alternative cut-offs of 5 and 10 patients produced broadly similar findings, supporting the robustness of the main analysis (Supplementary Table S3); however, any categorization of a continuous measure results in some loss of information. The additional analysis treating institutional enrollment as a continuous variable was therefore performed as a sensitivity analysis.

The absence of a survival difference by institutional enrollment volume in the surgery group is notable, given the well-established volume–outcome relationship in esophageal surgery. Population-based analyses have consistently shown lower perioperative mortality and improved long-term survival at high-volume centers [8, 9]. However, within a rigorously controlled clinical trial framework with predefined operative standards and quality monitoring, such disparities may be attenuated. Although no statistically significant association between institutional trial enrollment and survival was observed in the surgery group, several surgical practice variables differed between the groups, including abdominal approach, reconstruction route, and extent of lymphadenectomy (with D3 dissection more frequently performed in High). These differences likely reflect institutional practice patterns and complicate the interpretation of unadjusted survival comparisons. Therefore, residual confounding cannot be excluded, and the absence of a statistically significant association should not be interpreted as evidence that institutional differences were eliminated by trial participation. Despite this difference in lymphadenectomy extent, no clear difference in survival outcomes was observed between groups, with crossing survival curves noted during follow-up. These findings are consistent with the clinical equipoise that led to the development of JCOG2013, which aims to further evaluate the clinical significance of the extent of lymphadenectomy in this population [16].

In the CRT group, selected toxicities differed between the enrollment groups, but the direction of these differences was not consistent. All-grade acute esophagitis was more frequent in High, whereas radiation dermatitis and myocardial ischemia or infarction were more frequent in Low. These findings may reflect multiple factors, including differences in supportive care, toxicity assessment, patient characteristics, radiotherapy planning, or chance. Because no patient-level cardiovascular risk factors, radiotherapy dosimetric parameters, protocol-compliance assessments, or institution-specific quality-assurance data were available for this analysis, the observed differences cannot be attributed to differences in radiotherapy quality or standardization. Although toxicity assessment schedules were protocol-defined, some inter-institutional variation in the grading of subjective symptoms may still occur. Radiotherapy protocol deviations have been associated with inferior outcomes in cooperative group trials [10, 11]. Moreover, in esophageal cancer, cardiac radiation dose has been correlated with cardiac events and OS [12]. Myocardial ischemia or infarction occurred in only five patients, all in the Low group. Given the small number of events, the multiple exploratory AE comparisons, and the absence of baseline cardiovascular and cardiac dosimetric data, this observation should be regarded as hypothesis-generating. Further studies incorporating pretreatment cardiovascular risk, detailed cardiac dose-volume parameters, radiotherapy technique, and protocol-compliance data are needed to determine whether this finding is reproducible. JCOG0502 specified surgical and CRT procedures in the protocol and incorporated centralized data management, ongoing monitoring, audits, and review of protocol deviations to promote protocol compliance and data quality. However, the present analysis did not include institution-level measures of protocol adherence or radiotherapy quality, and therefore cannot determine whether these systems reduced inter-institutional variation in treatment delivery. Because JCOG0502 did not include elective nodal irradiation, its radiation fields were simpler and more standardized than in trials such as JCOG1904, where more complex target volume delineation may increase inter-institutional variability, underscoring the need for rigorous quality assurance in such settings [17].

This study has several limitations. First, it was an exploratory post hoc analysis and was not powered to demonstrate equivalence or to exclude clinically meaningful differences between institutions. Second, as discussed above, institutional trial enrollment is not equivalent to institutional case or procedural volume. Third, although baseline patient characteristics were generally comparable, several surgical practice variables differed between groups; therefore, residual confounding cannot be excluded. Fourth, the dichotomization of institutional enrollment at the median resulted in loss of information, although alternative cut-off analyses and the continuous-variable sensitivity analysis were performed to assess robustness. Fifth, patient-level cardiovascular risk factors, radiotherapy dosimetric data, institution-specific quality-assurance data, and detailed protocol-compliance measures were unavailable. Finally, multiple exploratory comparisons were performed without adjustment for multiplicity, and the toxicity findings may have arisen by chance. Accordingly, all findings should be interpreted as exploratory and hypothesis-generating.

## Conclusions

In this exploratory analysis of patients with stage I ESCC enrolled in JCOG0502, no clear association was identified between institutional trial enrollment volume and OS or PFS in either the surgery or CRT group. However, clinically meaningful differences could not be excluded. Differences in selected surgical practices and toxicities, including myocardial ischemia, should be regarded as hypothesis-generating and do not establish differences in treatment quality or CRT standardization. 

## Ethical statement

This study was conducted in accordance with the Declaration of Helsinki and the Ethical Guidelines for Medical and Health Research Involving Human Subjects in Japan. The original JCOG0502 trial protocol was approved by the institutional review board of each participating institution. Written informed consent was obtained from all participants before enrollment in the original trial.

## Supplementary Information

Below is the link to the electronic supplementary material.

Supplementary Material 1

Supplementary Material 2

## Data Availability

Individual participant data that underlie the results reported in this article, after de-identification, will be shared if investigators from the Japan Esophageal Oncology Group of JCOG are identified for this purpose and their proposed use of the data has been approved. Proposals should be directed to kenkato@ncc.go.jp. The data will be available to achieve the aims in the approved proposal.
