# Supplementary material for: Institutional enrollment volume and clinical outcomes after surgery or chemoradiotherapy for clinical stage I esophageal squamous cell carcinoma: a supplementary analysis of JCOG0502
Source: Esophagus. 2026 Aug 27;23(4):770–81. doi: 10.1007/s10388-026-01244-w (PMC13723696; doi:10.1007/s10388-026-01244-w)
Supplement: Supplementary file 1 [file 10388_2026_1244_MOESM1_ESM.docx]

**Supplementary Figure S1.**

Distribution of institutional trial enrollment among the 37 institutions contributing to the eligible analysis cohort (N = 364). Institutions are ranked in descending order of enrollment; bars are colored according to the median-based classification used in the primary analysis (red, high-enrollment institutions, ≥13 patients, n = 9 institutions, 207 patients; navy, low-enrollment institutions, <13 patients, n = 28 institutions, 157 patients). Median institutional enrollment was 13 patients (IQR, 7–26; range, 1–71).

*Institution-level counts reflect the eligible analysis cohort (N = 364; 37 institutions), derived from the all-enrolled institutional roster (N = 379; 38 institutions, one of which enrolled 0 eligible patients) after subtracting the 15 excluded patients (2 non-protocol treatment, 13 ineligible) according to their institution of enrollment.*
