# Supplementary material for: Institutional enrollment volume and clinical outcomes after surgery or chemoradiotherapy for clinical stage I esophageal squamous cell carcinoma: a supplementary analysis of JCOG0502
Source: Esophagus. 2026 Aug 27;23(4):770–81. doi: 10.1007/s10388-026-01244-w (PMC13723696; doi:10.1007/s10388-026-01244-w)
Supplement: Supplementary file 2 [file 10388_2026_1244_MOESM2_ESM.docx]

**Supplementary Methods: Radiotherapy Technique**

Radiotherapy was delivered using megavoltage equipment with a photon energy of 6 MV or greater and a source-to-surface distance or source-to-axis distance of at least 100 cm. The gross tumor volume comprised the primary tumor and any intramural metastases. The extent of the primary tumor was defined by endoscopy with Lugol staining, with reference to esophagographic findings, whereas intramural metastases were defined based on endoscopic findings with Lugol staining. The clinical target volume for the primary tumor and intramural metastases was generated by adding a 2-cm cranio-caudal margin to each corresponding gross tumor volume. Elective nodal irradiation was not permitted.

The planning target volume was generated by adding margins of approximately 0.5–1 cm laterally and 1–2 cm cranio-caudally to the clinical target volume to account for respiratory motion and setup variability. After accounting for dose fall-off, the typical distance from the tumor edge to the field edge was approximately 1.0–1.5 cm.

A total dose of 60 Gy in 30 fractions was delivered, with a planned treatment duration of 42 days and an allowable total treatment duration of up to 63 days. No planned treatment break was permitted. If irradiation could not be delivered because of a holiday or similar reason, treatment was postponed to the next treatment day without modification of the prescribed dose.

For tumors of the mid- or lower-thoracic esophagus, three- or four-field techniques were recommended where feasible to reduce cardiac exposure. The number of fields used for upper-thoracic tumors was determined at each institution. All planned fields were required to be treated at every fraction, and treatment using only one field of a multifield plan was not permitted.

In the transverse plane containing the reference point, the dose to the planning target volume was required to be within 95–110% of the prescribed dose. Three-dimensional dose calculations based on planning CT were required for multifield techniques. Heterogeneity and lung corrections were not used for monitor-unit calculations.

Endoscopic clipping at the cranial and caudal margins of the primary tumor and intramural metastases was recommended before treatment planning and was mandatory when tumor localization was difficult or when pretreatment esophagography had not been performed. When clipping was performed, placement within 1 cm of the tumor margins was recommended. Treatment planning was based on CT or radiographic simulation incorporating esophagographic findings, clip positions, and CT findings. Verification imaging was required at treatment initiation and whenever the treatment technique was changed; electronic portal imaging devices were permitted for this purpose.

**Supplementary Table S2.**

Association between institutional trial enrollment volume (continuous variable) and survival outcomes, estimated as the hazard ratio per five-patient increase in institutional enrollment.

| **Endpoint** | **Cohort** | **N** | **HR per 5-patient increase (95% CI)** |
| --- | --- | --- | --- |
| PFS | Surgery | 201 | 0.97 (0.94–1.00) |
| PFS | CRT | 162 | 0.99 (0.97–1.01) |
| OS | Surgery | 201 | 0.97 (0.94–1.00) |
| OS | CRT | 162 | 0.99 (0.96–1.02) |

*CI, confidence interval; CRT, chemoradiotherapy; HR, hazard ratio; OS, overall survival; PFS, progression-free survival. Hazard ratios were estimated using Cox proportional hazards models treating institutional trial enrollment as a continuous variable.*

**Supplementary Table S3.**

Sensitivity analysis of the association between institutional trial enrollment volume and survival using alternative enrollment cut-offs (5 and 10 patients), compared with the primary median-based cut-off (13 patients).

| **Cohort** | **Cut-off (patients)** | **n, Low** | **n, High** | **OS HR (95% CI)** | **OS P value** | **PFS HR (95% CI)** | **PFS P value** |
| --- | --- | --- | --- | --- | --- | --- | --- |
| Surgery | 5 | 15 | 186 | 1.73 (0.42–7.17) | 0.45 | 2.33 (0.57–9.60) | 0.24 |
| Surgery | 10 | 64 | 137 | 0.91 (0.48–1.73) | 0.77 | 0.94 (0.53–1.68) | 0.84 |
| Surgery | 13 (median, primary) | 97 | 104 | 0.73 (0.40–1.35) | 0.32 | 0.71 (0.41–1.23) | 0.22 |
| CRT | 5 | 12 | 150 | 1.00 (0.31–3.25) | 1.00 | 0.94 (0.34–2.60) | 0.91 |
| CRT | 10 | 48 | 114 | 1.02 (0.52–2.00) | 0.95 | 1.16 (0.70–2.03) | 0.59 |
| CRT | 13 (median, primary) | 59 | 103 | 1.12 (0.59–2.13) | 0.73 | 1.17 (0.70–1.97) | 0.55 |

CI, confidence interval; CRT, chemoradiotherapy; HR, hazard ratio (High vs Low institutional trial enrollment); OS, overall survival; PFS, progression-free survival. HR and 95% CI were estimated using Cox proportional hazards models; P values are two-sided Wald-type.
